# Biology and ecology of the Oriental flower-breeding *Drosophila elegans* and related species

**DOI:** 10.1080/19336934.2022.2066953

**Published:** 2022-05-01

**Authors:** Yuki Ishikawa, Masahito T. Kimura, Masanori J. Toda

**Affiliations:** a Graduate School of Science, Nagoya University, Nagoya, Japan; b Hokkaido University Museum, Hokkaido University, Sapporo, Japan

**Keywords:** Adaptive evolution, behavioural evolution, flower visiting, territoriality, colour polymorphism, sexual dimorphism, courtship display, mating behaviour, life-history trait, chromosome

## Abstract

Animals adapt to their environments in the course of evolution. One effective approach to elucidate mechanisms of adaptive evolution is to compare closely related species with model organisms in which knowledge of the molecular and physiological bases of various traits has been accumulated. *Drosophila elegans* and its close relatives, belonging to the same species group as the model organism *D. melanogaster*, exhibit various unique characteristics such as flower-breeding habit, courtship display, territoriality, sexual dimorphism, and colour polymorphism. Their ease of culturing and availability of genomic information makes them a useful model for understanding mechanisms of adaptive evolution. Here, we review the morphology, distribution, and phylogenetic relationships of *D. elegans* and related species, as well as their characteristic flower-dependent biology, food habits, and life-history traits. We also describe their unique mating and territorial behaviours and note their distinctive karyotype and the genetic mechanisms of morphological diversity that have recently been revealed.

## Introduction

The genus *Drosophila*, one of the most famous Diptera lineages, currently includes 1,262 species that are distributed throughout the world (DrosWLD-Species 2021; Available from: https://bioinfo.museum.hokudai.ac.jp/db/). They have adapted to a variety of environments from tropical to subarctic and from rainforests to deserts. One of these species, *D. melanogaster*, has long been studied as a model organism for genetics, developmental biology, and neurobiology. The research history of this species has led to the accumulation of knowledge regarding the molecular and physiological mechanisms of various traits. Recent remarkable advances in genome sequencing and editing have allowed us to apply this knowledge to other related *Drosophila* species, whose fascinating characteristics and natural history have been overlooked for a long time, to understand the molecular and physiological mechanisms of adaptive evolution.

One such species, *D. elegans*, shows various unique characteristics, such as flower-dependent life history, unique courtship display, territoriality, distinct sexual dimorphism, and colour polymorphism. Its ease of rearing in laboratory conditions, as well as the close relatedness to *D. melanogaster* (belonging to the same species group) and availability of whole-genome information, makes it a promising model for unravelling the molecular and physiological mechanisms of adaptive and behavioural evolutions. However, available knowledge of *D. elegans* is sparse and has so far not been synthesized. Thus, in this article, we will comprehensively review the basic biology and ecology of *D. elegans*, as well as the genetic mechanisms of some characteristics, which have recently been elucidated, and discuss directions for future research.

## Systematics, morphology, and distribution

**Figure 1.**
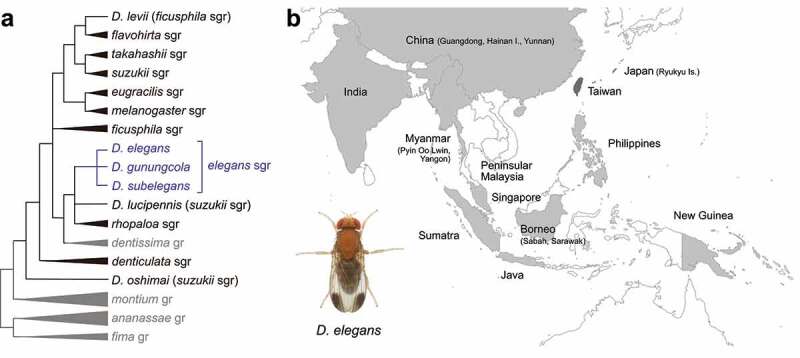
Phylogeny and distribution of *Drosophila elegans* and related species. (a) Phylogenetic tree of the *melanogaster* and related species groups, integrated from robust tree topologies inferred in previous molecular phylogenetic studies [1–5]. (b) Geographic distribution of *D. elegans*. Texts and filled areas indicate where *D. elegans* has been collected (light grey for brown morph, dark grey for black morph). Data from DrosWLD-Species (https://bioinfo.museum.hokudai.ac.jp/db/, DATA ID: 76707) [6–12]. A photo shows habitus (dorsal view) of a male of the brown morph type of *D. elegans*. This is reused with the kind permission of Dr. Nicolas Gompel (Image source: http://gompel.org/wp-content/gallery/drosophilidae/Drosophila-elegans-HK-iso1-genome-male-1x125-dorsal-enhanced.jpg). CC BY-NC-SA 3.0.

*D. elegans* belongs to the *elegans* species subgroup in the *melanogaster* species group, along with *D. neoelegans, D. sahyadrii, D. subelegans*, and *D. gunungcola*. The *elegans* subgroup is considered to form a clade together with the *rhopaloa* species subgroup and *D. lucipennis* (now placed in the *suzukii* species subgroup) (Figure 1(a)) [1–4]. This clade is placed as the sister to the *dentissima* species group, together comprising a lineage which relatively early branched off within the *melanogaster* group sensu stricto (excluding the *ananassae* and *montium* species subgroups) [5].

*D. elegans* was first described from the Philippines by Bock and Wheeler [6], but later found to be distributed over a wide range of the Oriental region and a part of the Australasian region: Ryukyu Islands in Japan, Taiwan, China, Philippines, Borneo, Sumatra, Java, Peninsular Malaysia, Singapore, Myanmar, India, and New Guinea (Figure 1(b)) [6–12] (DrosWLD-Species, DATA ID: 72261). However, the other members of the *elegans* subgroup are more restricted in their geographic distributions: *D. neoelegans* has been recorded from West Bengal of India and Yunnan of China [13,14] (DrosWLD-Species, DATA ID: 76837); *D. sahyadrii* only from Western Ghats of India [15] (DrosWLD-Species, DATA ID: 76856); *D. subelegans* only from Sri Lanka [16] (DrosWLD-Species, DATA ID: 77080); and *D. gunungcola* only from West Sumatra and Java in Indonesia [10] (DrosWLD-Species, DATA ID: 77187). *D. gunungcola* is distributed from middle to high altitudes on mountains, often in sympatry with *D. elegans* that is distributed from lowlands to highlands (Table S1) [10,17].

**Figure 2.**
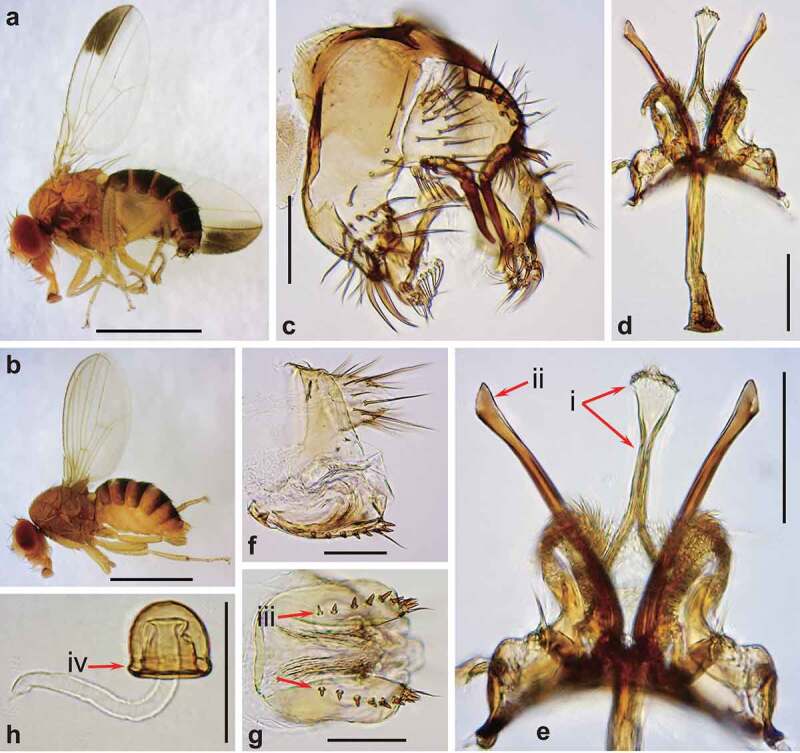
Morphology of *Drosophila elegans* brown morph. (a) Male habitus (lateral view). (b) Female habitus (lateral view). (c) Periphallic organs (caudolateral view). (d) Phallic organs (ventral view). (e) Ditto. (f) Female terminalia (lateral view). (g) Ditto (ventral view). (h) Spermatheca (lateral view). Diagnostic characters are indicated with red arrows: (i) aedeagus medially very narrow, apically expanded and truncate with nearly flat margin in ventral view; (ii) posterior elongation of pregonite apically slightly expanded triangularly; (iii) hypogynial valve (oviscapt) with teeth arranged in a single row on ventral margin; and (iv) spermathecal capsule as long as wide, with basal collar. Scales: 1 mm in a and b, 0.1 mm in c–h.

**Figure 3.**
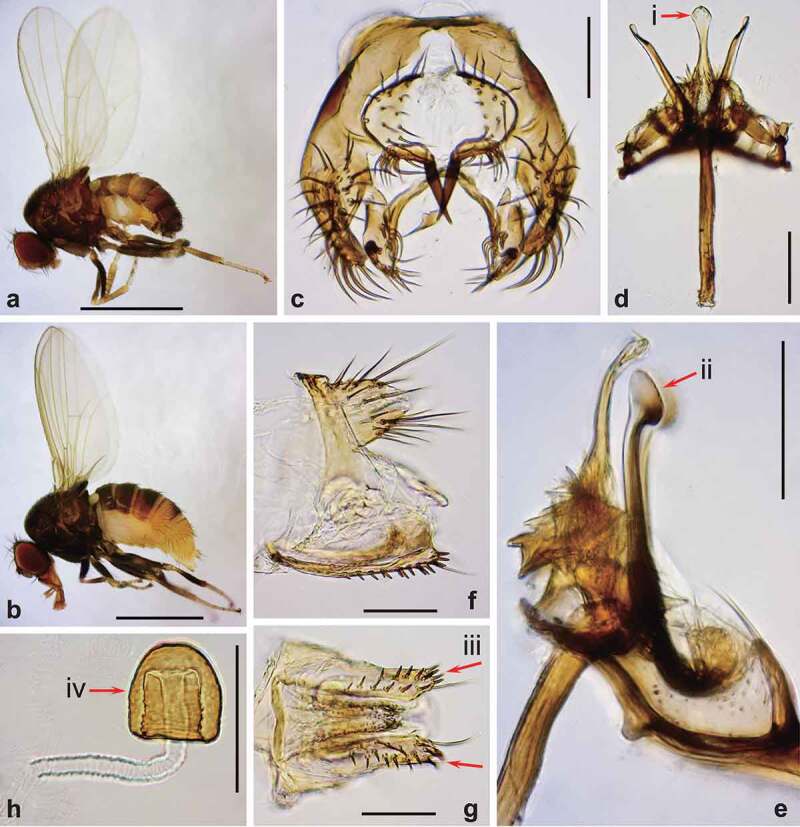
Morphology of *Drosophila gunungcola.* (a) Male habitus (lateral view). (b) Female habitus (lateral view). (c) Periphallic organs (caudal view). (d) Phallic organs (ventral view). (e) Ditto (lateral view). (f) Female terminalia (lateral view). (g) Ditto (ventral view). (h) Spermatheca (lateral view). Diagnostic characters are indicated with red arrows: (i) aedeagus apically expanded, with convex distal margin in ventral view; (ii) posterior elongation of pregonite apically expanded roundly; (iii) hypogynial valve (oviscapt) with teeth arranged in a few irregular rows on ventral margin; and (iv) spermathecal capsule longer than wide, without basal collar. Scales: 1 mm in a and b, 0.1 mm in c–h.

Males of *D. elegans* are well-distinguished by their wings with apical black patches (Figure 2(a)), sex combs in transverse rows on the first 3 tarsal segments, and deep-orange testes conspicuous through the ventral abdominal wall [6]. In both sexes, Malpighian tubules are orange, also visible through the ventral abdominal wall. Body sizes are similar to *D. melanogaster* (about 2.5 mm in males, and about 2.7 mm in females) [6], with significant natural variation [18,19]. Although most species in the *elegans* subgroup can be distinguished by detailed structures of male terminalia, the following two pairs of species are unclear in morphological differences of genital structures, based on their original descriptions and illustrations: *D. elegans*/*D. neoelegans* and *D. sahyadrii/D. subelegans* [6,13,15,16]. The species status should be re-examined for these pairs. Aiming at such a taxonomical revision of this species subgroup in the future, we present here microphotographs of the male and female terminalia for *D. elegans* (Figure 2) and *D. gunungcola* (Figure 3). In addition, for the last three species there is no or little information on their biology, ecology, or physiology, which should be complemented in future studies.

*D. elegans* shows intraspecific colour polymorphism, in which brown and black morphs are recognized (see **Genetic mechanisms of phenotypic divergence between *D. elegans* and *D. gunungcola***). The brown morph is widely distributed in the Asian continental area, the Greater Sunda Islands, Philippines, and New Guinea, while the black morph is restricted to Ryukyu Islands and Taiwan (Figure 1(b)) [6,8,9,12]. These morphs can be crossed, at least in a laboratory condition, though showing incipient reproductive isolation (see **Reproductive isolation**). No inter-morph differences in other morphological characters than the body colour have been reported, but behavioural and physiological traits are significantly diversified between the morphs [8].

## Flower breeding and food resources

**Figure 4.**
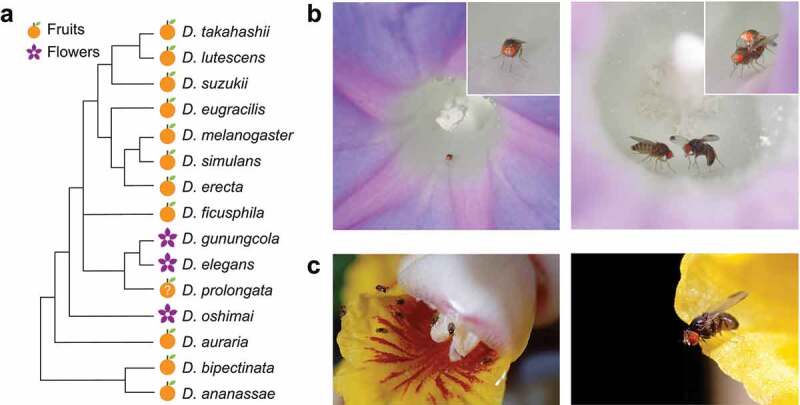
Breeding sites of the *Drosophila melanogaster* species group, and flower-visiting and courtship behaviours of *D. elegans.* (a) A phylogenetic tree based on the hypothesis shown in Figure 1(a), with breeding sites of the *melanogaster* and related species groups [7,10,20–24]. Symbols of fruits and flowers indicate the major breeding sites. Detailed information with references is available in Figure S1. (b) A *D. elegans* male staying in a flower of the host plant *Ipomoea indica* (left). The inset shows an enlarged image. A male of *D. elegans* courting a female with a wing display in a flower of *I. indica* (right). The inset shows successful copulation of a pair. The photos are captured by Dr. Ryoya Tanaka on Okinawa main Island. (c) *D. elegans* gathering in a flower of the host plant *Alpinia zerumbet*. The photos are captured by Dr. Takao Yoshida on Iriomote Island, and shared through Japan Drosophila Database (http://www.drosophila.jp/jdd/index_en.html).

*D. elegans* and related species (*D. gunungcola* and *D. sahyadrii*) are mostly found and/or emerge from flowers (Figure 4, S1; Table S1) [8,11,12,15,17]. In Iriomote Island (southern Japan), many *Drosophila* species have been observed to breed on fruits, but *D. elegans* has been recorded only from *Ipomoea* flowers [7], although a few individuals of *D. elegans* have once been collected by bait traps in non-flowering season in Taiwan (Kimura, unpublished data). A similar habit was observed for *D. sahyadrii* in the Western Ghats, southern India: this species was never attracted to banana-bait traps but collected exclusively from *Ipomoea* flowers [15]. These observations indicate that *D. elegans* and related species are flower-feeders, while most species of the *melanogaster* group are generalist fruit-feeders (Figure 4(a), S1). Ecological, behavioural, and physiological studies of the *elegans* subgroup have been conducted only on *D. elegans* and *D. gunungcola*.

So far, breeding of *D. elegans* and *D. gunungcola* has been predominantly reported from *Ipomoea* flowers, particularly *I. cairica* and *I. indica* (Figure 4(b); Table S1) [7,8,10–12,17]. However, not all *Ipomoea* species are suitable for their breeding. In Iriomote Island, for example, *D. elegans* has been frequently observed breeding on *I. indica* flowers but has never exploited *I. pes-caprae* flowers [7]. Yoshida et al. [11] further reported that *D. elegans* did not visit *I. pes-caprae* flowers even though it frequently visited neighbouring *I. indica* flowers, suggesting that *I. pes-caprae* flowers are unsuitable for *D. elegans* to use them as breeding sites. On the other hand, *D. elegans* breeds on flowers of the shell ginger *Alpinia zerumbet* (Figure 4(c)), which was misidentified as *Curcuma* (misspelled ‘*Cucurma*’) *domestica* in Yoshida et al. [11], on this Island. Why and how *D. elegans* selects *I. indica, I. cairica*, and *A. zerumbet* flowers for breeding is not known. A common feature of these *Ipomoea* and *Alpinia* flowers is short longevity; individual flowers of these species bloom only for one or two day(s) and then fall on the ground. Such short-lived flowers may produce less defensive substances and attract fewer consumers, thus implying that they are toxin- and competitor-free resources for a few consumers specialized to exploit them [25]. However, flowers of the angel’s trumpets *Brugmansia suaveolens* and *B. candida*, of which individual flowers contain alkaloid toxins and bloom for several days, were observed being visited and used as breeding sites by *D. gunungcola* at high altitudes in Indonesia [17]. *D. elegans* also visits these flowers but its breeding on them has never been confirmed [17]. It is either unknown why and how *D. gunungcola* exploits these flowers. *D. elegans* and *D. gunungcola* could locate these flowers utilizing the flower colour, size, shape, and/or chemical components as cues. To understand how they locate flowers, systematic studies that incorporate knowledge of the insect sensory system are required.

Female flies of *D. elegans* and *D. gunungcola* lay eggs on blooming flowers and the offspring larvae develop in the fallen decayed flowers. Decayed flowers differ in nutritional conditions from fermented fruits on which larvae of generalist species feed; the former contains fewer carbohydrates and more proteins (i.e. high P:C ratio), whereas the latter contains more carbohydrates and fewer proteins [26]. In experiments using artificial fly foods, the developmental performance of *D. elegans* larvae was drastically decreased when reared on foods containing high carbohydrates but low proteins, on which the generalist *D. melanogaster* and *D. simulans* maintained their normal development [26]. In addition, high-carbohydrate and low-protein foods exacerbate male life span and female egg production in *D. elegans* [27]. This suggests that *D. elegans* has tuned its metabolic pathways to exploit flowers with low carbohydrates and high proteins.

Unlike larval food, it is difficult to identify adult food sources in natural habitats. It is likely that adults of *D. elegans* also obtain nutrients from flowers because they sometimes lick the surface of petals, stamen, and pistil of flowers (Ishikawa, unpublished). They may feed on nectar, pollen, or microorganisms growing on flowers.

Further, *D. elegans* females and larvae show unique oviposition and feeding behaviours; females lay eggs on the surface of stamens and petals unlike females of most other *Drosophila* species that embed their eggs into the substrates, and larvae crawl on the surface of stamens and petals and seem to feed by licking or whittling. Even on laboratory *Drosophila* medium, females do not embed their eggs. On the other hand, larvae crawl into the laboratory medium, though their development is dependent on the hardness of medium; their viability is improved by reducing the agar concentration of the medium (Table 1, Ishikawa unpublished data). Although fly stocks collected from the field are initially less adapted to the ordinarily hard laboratory medium, their performance is gradually improved generation by generation.

**Table 1.** Tested artificial fly foods varying in ingredient ratios and the breeding performance of *Drosophila elegans.*

Agarose (g/L)	Cornmeal (g/L)	Yeast (g/L)	Glucose (g/L)	Breeding condition	Note
8	40	45	50	fair	Food for *D. melanogaster*
8	10	11	25	poor	
6	40	45	12.5	fair	
6	40	45	25	fair	
6	20	22.5	25	good	
5	20	22.5	25	good	
4	20	22.5	25	excellent	

## Life-history traits, seasonality, and temperature tolerance

Knowledge of life-history traits, such as developmental period, life span, and egg-production period, as well as seasonality and temperature tolerance, is essential for understanding animal ecology. Such knowledge in natural habitats, however, is not yet sufficient in *D. elegans* and related species. Instead, phenotypes under experimental conditions have been investigated. When host flowers with eggs of *D. elegans* are collected from natural habitats and kept at a constant temperature of 25°C, eclosion of adult flies peaks 9–10 days after collection (Ishikawa unpublished), which is equivalent to that of *D. melanogaster* bred with normal fly food [28]. Even when grown on a cornmeal fly food, the developmental period is not very different from that grown on host flowers, i.e. about 10 days for *D. elegans* and about 11 days for *D. gunungcola* at 23°C [17], although the high-carbohydrate and low-protein diet interferes with the development (see **Flower breeding and food resources**) [26]. At lower temperatures, the developmental period becomes longer; approximately 20 days in both *D. elegans* and *D. gunungcola* at constant 18°C. Notably, the strength of the temperature effect varies by sex [17], suggesting temperature-dependent sex-specific modification of larval development.

As for *D. elegans* adults, the median life span is approximately 40 to 50 days in males and 65 days in females when reared on fly food at constant 25°C, within the range of *D. melanogaster* and other related species [27]. Considering that daily temperature fluctuation drastically elongates the life span of *D. melanogaster* [29], the natural life span of adult *D. elegans* might be much longer. The nutrient ratio of fly food affects the adult life span in a sex-specific manner; high-carbohydrate and low-protein food decreased male life span, while no effect was found on females [27].

The number of eggs laid by *D. elegans* is extremely low compared to other generalist species (*D. melanogaster* and *D. simulans*); 40 to 100 eggs were laid by 4 females for 8 days, only 13% of those laid by *D. melanogaster* in similar conditions [27]. The egg size is slightly, but not extremely, bigger than that of *D. melanogaster*: Egg volume (mm^3^ × 10^−2^) = 1.27 ± 0.01 (mean ± SD) in *D. elegans*, 1.23 ± 0.03 (mean ± 95% confidence limits) in *D. melanogaster* [17,31]. Egg production peaks 1 to 2 days after copulation (approximately 4.5 to 8.5 eggs per female), then declines for 10 days (approximately 1 to 2 eggs per female), and remains at a low level for at least the next 10 days [27,32]. Considering that copulation inhibits re-mating for at least one to several weeks (see **Re-mating**), copulation with virgin females may be extremely important for male reproductive success.

The population densities of *D. elegans* and *D. gunungcola* fluctuate seasonally in natural habitats. In Java, both species decrease their densities during the dry season, May to October [17]. Since *I. indica* blooms even during the dry season, factors other than the host-flower abundance may affect the population density. The seasonality of *D. elegans* has not yet been investigated in regions different in temperature, humidity, and host-flower density. Under experimental conditions, the lower and upper half lethal temperatures (LT_50_) for 24-h exposure are approximately 4 to 6.5°C and 33.5°C, respectively, in *D. elegans* [8]. Compared to *D. elegans, D. gunungcola* shows less tolerance for high temperature, but similar tolerance for low temperature [10]. Such temperature tolerance may also be a factor limiting the temporal and geographic distribution.

## Courtship behaviour

**Figure 5.**
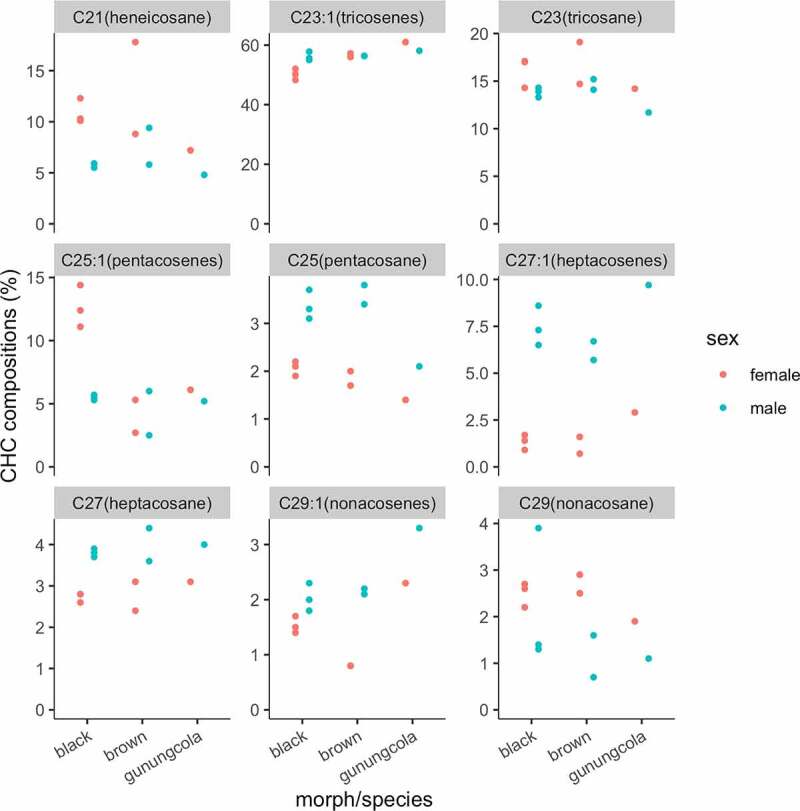
Cuticular hydrocarbons (CHCs) of *Drosophila elegans* and *D. gunungcola.* The CHC compositions (%) of black and brown morphs of *D. elegans* and *D. gunungcola*. Data from [42,43]. Each point indicates asingle data point from 200 flies.

In host flowers, males of *D. elegans* court to and copulate with females. Their courtship behaviour is well characterized by prominent wing displays (Figure 4(b), right). After tapping a female, the male circles in front of her, spreads his spotted wings horizontally, repeatedly waves his wings, and moves laterally to the female with abdominal bending to her [33–35]. The display of wing spots, which exist only in males, serves as a visual courtship signal; the closely related species *D. gunungcola*, which lacks the spots on male wings, does not show the wing display during courtship [35,36] (but also see [33] and **Genetic mechanisms of phenotypic divergence between *D. elegans* and *D. gunungcola***) Figure 5. Such co-evolution of wing spots and wing display has also occurred in other species of the *melanogaster* group (such as the *suzukii* subgroup) [37–40]. Many researchers have predicted that wing display contributes to male mating success, yet all statements made so far have no supportive experimental evidence [35].

Because of the prominence of the wing display, little attention has been paid to other courtship behaviours. Recently, courtship songs were first reported in *D. elegans* and *D. gunungcola* (Extended Data Figure 6(a) in [41]). Although no systematic analysis has been performed, at least two types of courtship songs seem to be species-specific: pulse-like song is produced by *D. elegans* but burst-like song by *D. gunungcola*. Further studies are required to examine the behavioural function of these songs in relation to copulation success.

**Figure 6.**
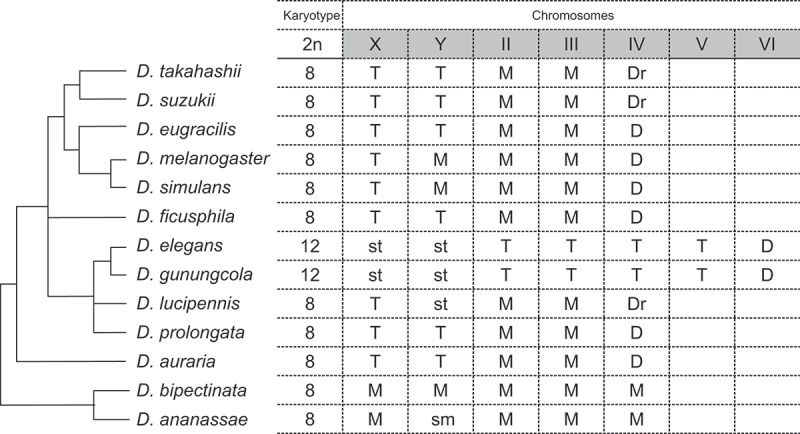
Karyotype and chromosome types of the *Drosophila melanogaster* and related species groups. A phylogenetic tree with the karyotype and chromosome types [1,13,35,46]. Chromosome types are classified into metacentric (M), submetacentric (sm), subtelocentric (st), telocentric (T), dot (D), and rod-like dot (Dr). In , Muller’s elements A, B, C, D, E, and F correspond to the chromosomes X, 2L, 2R, 3L, 3R, and IV, respectively.

## Reproductive isolation

*D. elegans* and *D. gunungcola* are distributed sympatrically and use flowers of the same host plant species as mating arenas (see **Flower breeding and food resources**), suggesting the differentiation of mate recognition system between these species. Indeed, in the male-choice test with one male and two females, males of both species preferentially courted to conspecific females rather than heterospecific ones, although they occasionally showed courtship to heterospecific females as well [42]. Since no interspecific copulation was observed under this experimental condition, the mate recognition system between these species is likely strong enough to contribute to premating isolation. However, when these species were crossed in the no-choice method (i.e. *D. elegans* ♂ × *D. gunungcola ♀* or *D. elegans ♀* × *D. gunungcola* ♂), both reciprocal crosses produced F_1_ hybrid progenies; such F_1_ hybrid females were typically fertile without any morphological abnormality, while F_1_ males were completely sterile and often show morphological abnormality in abdominal tergites [34]. Thus, the post-mating isolation is partial between these two species.

Within *D. elegans*, incipient reproductive isolation is present between the two morphs, black and brown [8,43]. In both male- and female-choice tests, assortative courtship and/or mating were observed between males and females of the same morph, although the isolation index was sometimes insignificant. These results suggest that the pre-mating isolation is present to some extent between these two morphs. However, a weak and asymmetrical post-mating isolation was observed between the brown and black morphs: intermorph crosses produced fertile F_1_ males and females whose viability was not significantly lower than that of parental flies, but the viability was significantly lowered in F_2_ progenies of crosses between females of the brown morph and males of the black morph [8]. The body colour phenotypes were split into brown, intermediate, and black in the ratio of approximately 1:2:1 in F_2_ progenies of crosses between the brown and black morphs [8]. In the female-choice [8] and male-choice [43] tests using the brown and black F_2_ progenies, neither the parental brown nor black morph flies (males and females) discriminated the F_2_ mates by their colours, indicating that the body colour is not an important cue for mate discrimination.

Cuticular hydrocarbons (CHCs) are strong candidates to provide signals for discriminating the sex, colour morph, and species, as in most *Drosophila* species. Although *D. elegans* has been reported to have ‘sexually monomorphic chemical profiles’ (i.e. the same compounds are found in both sexes) [45], the amount of CHCs varies by sex, morph, and species (Figure 5) [42,43]. For sexual differences, the compositions of pentacosane and heptacosenes are higher in males than in females in both morphs of *D. elegans. D. gunungcola* also shows a similar trend, although the difference in pentacosane is not so obvious. In females, although there is no clear difference in the CHC composition, the molecular composition of pentacosenes differs between the species [42]. For intraspecific variation between the two morphs of *D. elegans*, the composition of pentacosenes is sexually dimorphic in the black morph, while no difference is found in the brown morph. These CHC differences probably contribute to the reproductive isolation between *D. elegans* and *D. gunungcola* and between the colour morphs of *D. elegans*, although further comprehensive studies, including molecular identification and quantitative and functional analysis of each component, will be required in the future.

## Re-mating

Although re-mating is observed in many *Drosophila* species, it has not been investigated if wild *D. elegans* flies mate multiple times in natural conditions. Under laboratory conditions, the first copulation of *D. elegans* inhibits the second copulation for over 10 days [32]. This inhibition does not occur when the copulation is interrupted before sperm transfer. The re-mating ratio gradually increases thereafter. The recovery speed significantly varies among the strains; HK strain (brown morph) originated from Hong Kong shows fast and high recovery (80% after 15 days from the first copulation), while OH strain (black morph) originated from Okinawa shows slow and low recovery (10% after 15 days from the first copulation). *D. gunungcola* is more difficult to re-mate; no re-mating was observed for 12 days after the first mating [19]. However, it is not clear to what extent this trait of each strain reflects the nature of the wild population. Unfortunately, the experimental conditions were only briefly described in [32]; the temperature was 23°C, but the photoperiod was unknown. It is known that temperature and photoperiod significantly affect the courtship activity of *Drosophila*. Because outside temperatures on the main Island of Okinawa exceed 23°C from April to November, the re-mating recovery process may be affected by these higher temperatures in the natural condition than that in the experimental condition. To consider the meaning of re-mating in *D. elegans*, we further need to take account of the adult lifespan and egg-production period under the natural condition (see **Life-history traits, seasonality, and temperature tolerance**), although such knowledge is not yet sufficient.

## Territoriality

Males of *D. elegan*s and *D. gunungcola* have been reported to establish a mating territory on individual *Ipomoea* flowers; when a male visits a flower in which another male already resides, the resident male usually chases and expels the intruder [18,19]. This is a typical territory defence behaviour [44,47]. On the other hand, there is no evidence that females are territorial.

Battles between the resident and the intruder last for a few to several tens seconds in nature [19]. However, battles between laboratory-reared males sometimes last for more than 10 min [18]. This is probably because laboratory-reared males are less variable in body size than wild males. Indeed, the body size is one of the major factors that can affect the outcome of a battle in *D. elegans* at least under the experimental conditions; winners have significantly larger bodies than losers [18]. It has also been observed under sympatric situation of *D. elegans* and *D. gunungcola* in nature that males show higher aggression to conspecific males than to allospecific ones, suggesting that they distinguish the species in territorial behaviour [19].

Expression of territoriality changes with time of day [18]. Males start to occupy newly blooming *Ipomoea* flowers in the early morning, and the number of territorial individuals increases during the day. When the fly density becomes high in the evening, some males fail to expel invaders. Finally, territoriality breaks down, and many males and females stay relatively still in the same flower. These field observations suggest that circadian mechanisms possibly affect the aggressiveness of males, but no systematic investigation has been conducted so far.

## Karyotype, chromosomes and genome size

The karyotypes of *D. elegans* and *D. gunungcola* are 2n = 12, composed of four pairs of rod-shaped chromosomes, one pair of dot-like chromosomes, and a pair of sex chromosomes [35,46]. This is exceptional in the *melanogaster* group; many other species have a karyotype of 2n = 8 (Figure 6) [46,48]. The linkage groups correspond to the six Muller elements A–F, although many rearrangements are observed [35]. The four autosomes of *D. elegans* are subteleocentric, while two autosomes are metacentric in other species of the *melanogaster* group (Figure 6) [46]. These configurations suggest that dynamic karyotypic evolution, such as chromosomal fission, has occurred in the *elegans* subgroup. It is worth investigating in future studies whether such karyotypic evolution relates to any characteristic properties of the *elegans* subgroup.

The haploid genome size of *D. elegans* estimated by the flow cytometry is 0.20 ± 0.003 pg in males and 0.19 ± 0.007 pg in females (mean ± SD) [49]. These values remain within the range observed in the *melanogaster* species group, where 0.16–0.18 pg in *D. melanogaster*, 0.19–0.21 pg in *D. takahashii*, and 0.25–0.29 pg in *D. lucipennis* [49].

## Genetic mechanisms of phenotypic divergence between *D. elegans* and *D. gunungcola*

Several phenotypic traits such as wing spots, wing display, body colour, and temperature response have been differentiated between *D. elegans* and *D. gunungcola*. Recent advances in genome sequencing and genome editing enabled us to investigate the genetic and molecular mechanisms of phenotypic evolution in these species. In the last section, we review the recently unravelled mechanisms for inter- and intra-specific variations in wing spot, wing display, and body colour.

*D. elegans* males possess wing spots and perform wing displays, while *D. gunungcola* males are considered to have lost both traits [10, but see 33]. Such correlation of wing pigmentation and wing display is often found in the *melanogaster* group [30,36,40,50,51]. To understand the genetic mechanisms of this correlated phenotypic evolution, Massay et al. [33] performed precise quantitative trait locus (QTL) mapping of both traits. They identified a ~ 440 kb region of the X chromosome with a large effect on the wing-spot size, as well as two additional loci on Muller Elements C and E (Chromosome 2R and 3R in *D. melanogaster*, respectively) with smaller effects. This region of the X chromosome includes the candidate gene *optomotor-blind* (*omb*), which encodes a T-box-containing transcription factor [52,53] and plays a critical role in patterning of the *Drosophila* wing [54]. Indeed, *omb* is expressed in the wing hinge and distal wing tip 30 h after puparium formation both in *D. elegans* and *D. gunungcola* [33].

As for the loci contributing to divergence in wing displays, multiple significant QTL on the X chromosome, Muller Elements B and E, which behave approximately additively, were identified. Thus, the genetic basis of divergent wing displays seems to be more complex than that of wing spots. Importantly, the wing-spotless strains, in which a spotless allele derived from *D. gunungcola* was introgressed into *D. elegans*, performed wing displays indistinguishable from *D. elegans*. The authors also found that the spotless *D. gunungcola* males of a wild population in East Java performed wing displays, despite no wing display by males of the SK strain from Sukarami, West Sumatra, which was used in all previous studies [10,33]. These findings indicate the loci controlling the wing spots and wing displays are genetically separable, and these two traits evolved independently between these species. Although it remains unknown whether *D. gunungcola* males with/without wing displays are mixed in a wild population, these results suggest that the loss of wing spots predates the loss of male wing displays in this species.

As mentioned above, the brown and black morphs are known in *D. elegans* [8] (see **Systematics, morphology, and distribution**). On the other hand, all populations of *D. gunungcola* so far found have black bodies. Massey et al. [55] conducted QTL analysis using *D. gunungcola* and the brown morph of *D. elegans* and found that thorax and leg pigmentation was largely affected by two genomic regions, one on the X chromosome and the other on Muller Element E, which contained the candidate pigmentation genes *yellow* and *ebony* [55]. Indeed, *D. elegans ebony* null mutants, which are generated by CRISPR/Cas9 system, show dark black bodies like *D. gunungcola* [55]. Thus, as is often the case with other *Drosophila* species [56], the genes *yellow* and *ebony* likely contribute to the diversification of pigmentation between *D. elegans* and *D. gunungcola*. However, it has not yet been determined whether these are also responsible for the differentiation of body colour between the two morphs of *D. elegans*.

## Perspectives

In this review, we described the fascinating characteristics exhibited by *D. elegans* and related species, including flower-dependent life history with specialized feeding habits, courtship display, territoriality, sexual dimorphism, and colour polymorphism. Unlike many other insects, *D. elegans* and *D. gunungcola* are easy to rear under laboratory conditions, and genome editing techniques can be implemented. Indeed, genome editing combined with high-throughput sequencing technology has revealed the genetic mechanisms of the diversity of wing spots, courtship display, and body colouration of these species. Future expansion of this approach will reveal the molecular and physiological bases for adaptive evolution of various traits.

Comparative approaches using model organisms and closely related species provide us with new opportunities to elucidate the molecular and physiological basis of adaptive evolution with unprecedented resolution. Indeed, comparisons between *D. melanogaster* and closely related species have revealed the molecular mechanisms of the evolution of food-related olfactory preference, as well as the neural basis of the diversity of mating preference [57,58]. Comparative connectome of the microbivore nematode *Caenorhabditis elegans* and the predatory nematode *Pristionchus pacificus* revealed the divergence of neural wiring associated with feeding habitat [59]. Although a systematic comparison of genomes or connectomes between *D. elegans* and *D. melanogaster* has never been made, it will be useful to clarify the molecular and physiological bases behind the unique biology and adaptive evolution in the *elegans* subgroup.

One of the most interesting aspects of the biology of *D. elegans* is its flower-dependent life history. In particular, flower-visiting behaviour has independently evolved in various insect lineages and is essential to maintain the relationship between plants and insects in terrestrial ecosystems. Bees, such as honeybees (*Apis mellifera*) and bumblebees, are the most studied flower visitors, especially with regards to the relevance of colour vision and olfaction for floral recognition [60,61]. In the field, flies are also major flower visitors, and in some plant species and regions, they exceed the number of flower visits by bees [62]. Since bees and flies have acquired flower-visiting behaviour independently, a comparison of these systems can reveal the general principle of the mechanism for the evolution of floral visitation. Furthermore, *D. elegans* is capable of genome editing and transgenic generation, which is challenging in honeybees and bumblebees due to their long generation times and the difficulty of artificial crosses. Thus, the study of *D. elegans* provides an interesting model for unravelling the neural mechanisms of how insects recognize and visit flowers with their small brains.

Our ecological knowledge of *D. elegans* in the field is not yet sufficient to elucidate their flower-dependent life history and related characteristics. In particular, floral preferences and related life-history traits have only been studied in specific regions and seasons, and we, therefore, are far from comprehensive understanding. The high dependence of *D. elegans* on flowers may lead to diversification of flower preference adapted to flowering phenology, thus field exploration in areas with different floral phenology is worthy of investigation. If such local adaptation exists, it would be a compelling example of adaptive evolution and provides a great opportunity to understand the underlying molecular and physiological mechanisms. By combining ecological insights from the field with systematic comparisons of genomes and connectomes, as well as functional analyses by high-throughput sequencing and genome editing, *D. elegans* will provide an attractive model for elucidating the molecular and physiological bases of adaptive evolution.

## Supplementary Material

Supplemental Material
