# Supplementary material for: Biology and ecology of the Oriental flower-breeding Drosophila elegans and related species
Source: Fly (Austin). 2022 May 1;16(1):207–20. doi: 10.1080/19336934.2022.2066953 (PMC9067466; doi:10.1080/19336934.2022.2066953)
Supplement: Supplemental Material [file KFLY_A_2066953_SM9252.zip › figS1_20220424.pdf]

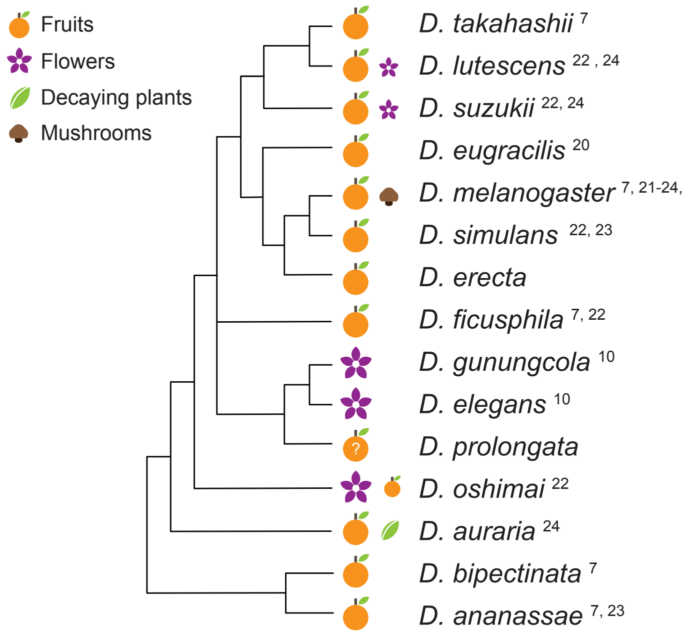

Fig.S1| **Breeding sites of the *Drosophila melanogaster* species group (detailed)**
A phylogenetic tree based on the hypothesis shown in Figure 1A, with breeding sites of the *melanogaster* group. Large and small symbols of fruits, flowers, decaying plants, and mushrooms indicate the major and minor breeding sites, respectively. The actual breeding sites of *D. prolongata* in the field are not clear, but they are likely to breed on fruits since they are attracted to banana-bait traps (Dr. Takashi Matsuo, personal communication). Superscript numbers indicate references: (7) Hirai et al. Entomol Sci. 2000, (10) Sultana et al. Entomol Sci. 1999, (20) Kimura and Suwito. J Nat Hist. 2012, (21) Kimura et al. Kontyû. 1977, (22) Mitsui et al. Entomol Sci. 2010, (23) Valadão et al. Ecol Entomol. 2019, (24) Shorrocks, In: The Genetics and Biology of Drosophila. Vol. 3b.1982.
